# Bearing-Foreign Material Deposition on Retrieved Co-Cr Femoral Heads: Composition and Morphology

**DOI:** 10.1155/2015/967278

**Published:** 2015-07-05

**Authors:** Nishant M. Tikekar, Anneliese D. Heiner, Thomas E. Baer, Karen M. Kruger, John J. Callaghan, Thomas D. Brown, John J. Lannutti

**Affiliations:** ^1^Department of Materials Science and Engineering, Ohio State University, 446 MacQuigg Laboratory, 105 W Woodruff Avenue, Columbus, OH 43210, USA; ^2^Department of Orthopaedics and Rehabilitation, University of Iowa, University of Iowa Hospitals and Clinics, 200 Hawkins Drive, 01008 JPP, Iowa City, IA 52242, USA; ^3^Department of Biomedical Engineering, University of Iowa, 1402 Seamans Center, Iowa City, IA 52242, USA

## Abstract

Bearing-foreign material deposition onto a femoral head can occur from contact with an acetabular shell due to dislocation, reduction, or subluxation. The purpose of this study was to comprehensively characterize deposit regions on retrieved cobalt-chrome femoral heads from metal-on-polyethylene total hip arthroplasties that had experienced such adverse events. The morphology, topography, and composition of deposition regions were characterized using macrophotography, optical profilometry, scanning electron microscopy, energy dispersive spectroscopy, and X-ray photoelectron spectroscopy. The deposit areas were relatively large, they were much rougher than the surrounding undamaged clean areas, and they displayed several distinct morphologies. Titanium alloy elements were the predominant constituents. Calcium and phosphorous were also detected within the deposit areas, in a composition that could nucleate abrasive hydroxyapatite. In addition, tungsten-rich particles, likely present as tungsten carbide, were observed on top of the titanium deposits. The increased roughness associated with these deposition features would be expected to accelerate damage and wear of the opposing liner and hence accelerate the development of osteolysis.

## 1. Introduction

Deposition of titanium or titanium alloy on a femoral head can roughen the surface and hence accelerate the wear of the opposing surface [1, 2], especially for metal-on-polyethylene implants. Titanium deposition on femoral heads has been reported in many retrieval series [1–4]. Such deposition can occur from contact of the head with a titanium-alloy acetabular shell, as a result of dislocation, closed reduction of a dislocation, extreme subluxation, liner wear-through, or liner dissociation. Titanium alloy transfer from an acetabular shell to a cobalt-chrome or alumina femoral head can occur with scraping contact at loads as small as 10 kg, whereas scratching damage to the head tends to occur only at larger loads [3]. Titanium alloy can also be transferred onto a femoral head from third-body debris within the bearing couple [4]. However, despite frequent visually-based qualitative descriptions in forensic assessments of retrieval femoral heads, the formal composition and topography of large transfer deposit areas have not been well characterized.

The presence of titanium (and other elements) transferred to a femoral head has often been identified by energy dispersive spectroscopy (EDS). Other commonly utilized techniques to characterize femoral head deposits are photography, micrography, scanning electron microscopy (SEM), and contact or optical profilometry (OP). The purpose of this study was to comprehensively and nondestructively characterize areas on retrieved cobalt-chrome femoral heads displaying visual and clinical evidence of deposition. Both qualitative and quantitative techniques were used to characterize these depositions in terms of morphology, topography, and composition.

## 2. Materials and Methods

**Figure 1:**
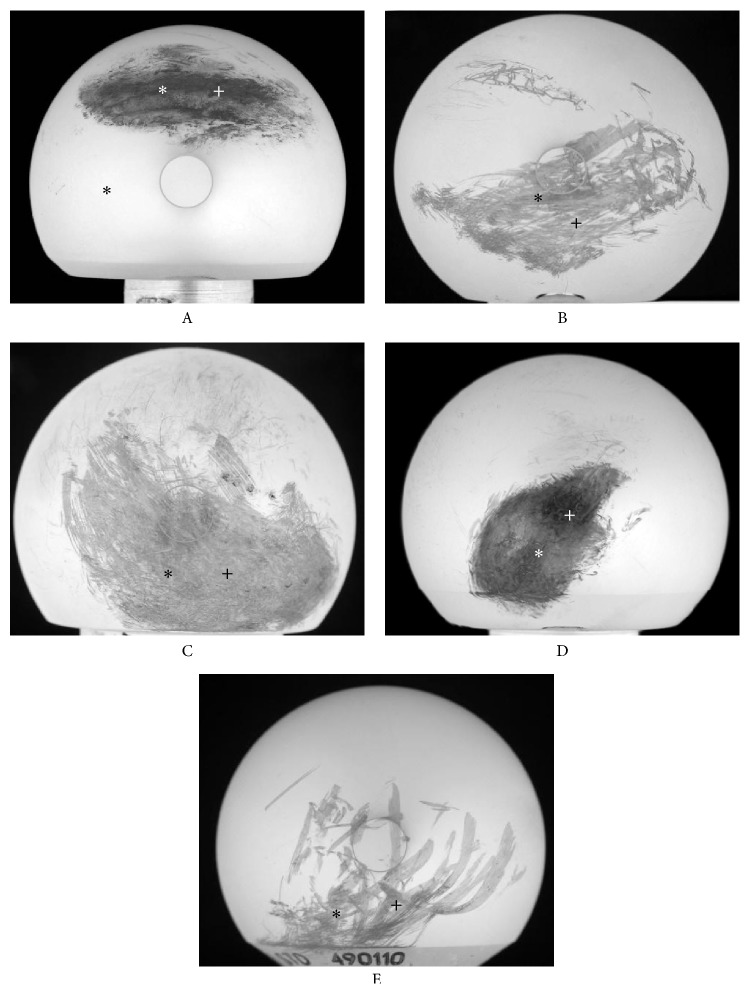
Diffused-light macroscopic images of the five femoral heads showing extensive bearing-foreign material deposits. The circles in the middle of images of heads A, B, C, and E delimit the reflection of the camera lens, which was eliminated by digital combination of photographs. The cross-mark (+) in each image identifies a corresponding site of SEM-based morphologic characterization in Figure 4, and the asterisks (*∗*) designate the sites of EDS evaluation in Figure 5.

Femoral heads from metal-on-polyethylene revision retrievals were used for this analysis. Five cobalt-chrome femoral heads from an International Review Board- (IRB-) approved retrieval collection of 199 total hip arthroplasty (THA) heads were selected on the basis of displaying representatively conspicuous evidence of transfer deposition (Figure 1). Sites for microscopic-level morphological and compositional analysis were identified using a diffused-light photographic technique that minimized ambient room reflectivity [5]. Purpose-designed fixturing was developed to allow spatial registration of the polar coordinates of macroscopically identified sites of interest with desired positions for microscopic-level scanning.

Clinical contexts for the five retrieved femoral heads were as follows.

**Figure 2:**
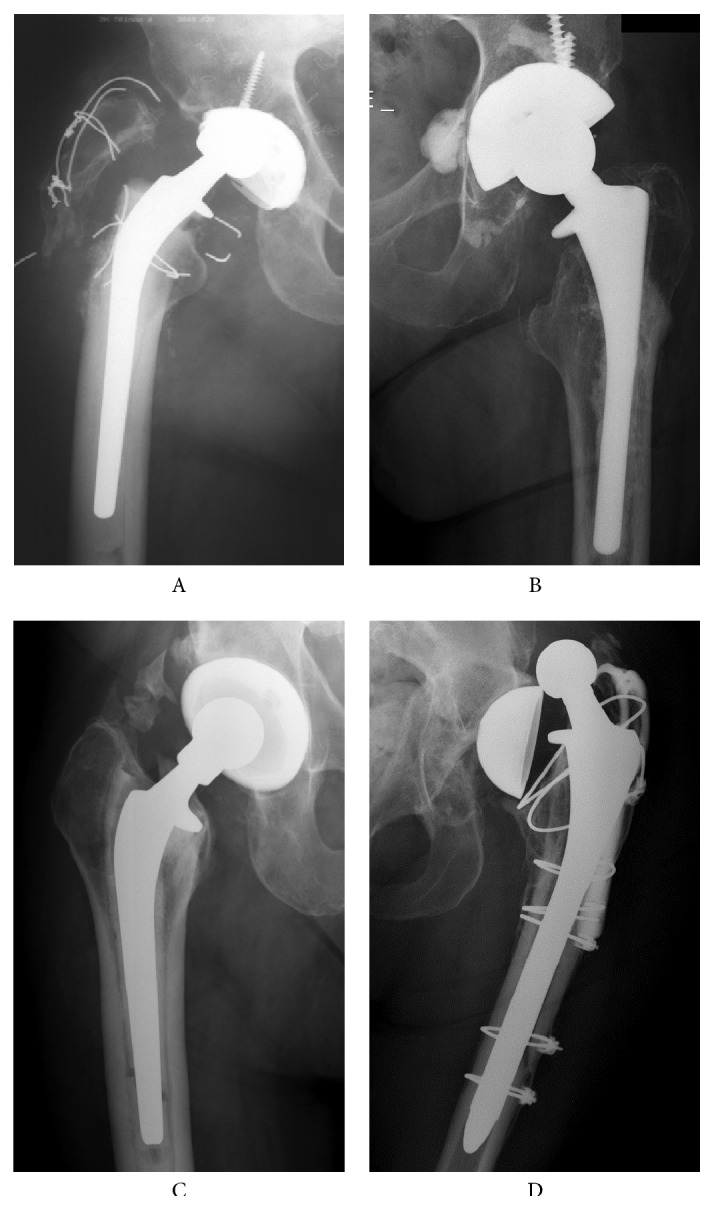
Radiographs from patients from whom heads A–D were retrieved. Radiographs of head E were not available.

*Head A*. A 76-year-old male presented with subluxation of his right hip 16 years after implantation (Figure 2A). He was noted to have had increasing acetabular liner wear over the preceding three years, but he had declined revision. He also had a trochanteric nonunion. After the subluxation was reduced, he elected to have revision surgery. Intraoperative findings included soft tissue metallosis. The liner was substantially worn and had undergone a rim fracture.

*Head B*. A 91-year-old female presented 18 months postrevision THA with a history of three dislocations of her hip within a three day interval, seven months previous to presentation. Radiographs (Figure 2B) demonstrated a loose femoral component and the stem was found to be loose at the time of revision. The liner had embedded debris and impingement damage that was less than 1 mm deep (Grade 2, Hospital for Special Surgery (HSS) scale) [6].

*Head C*. An active obese 48-year-old male, a tow truck operator, was initially revised for instability one month postprimary THA. Six years later, a second revision was performed for aseptic loosening and late (5 years post op) recurrent (six times) dislocation. Radiographs (Figure 2C) demonstrated loosening of the femoral component and a stable acetabular component. The femoral component was grossly loose at the rerevision surgery. The liner had embedded debris and impingement damage that was greater than 2 mm deep (Grade 4, HSS scale).

*Head D*. A 53-year-old female presented 4.5 years postrevision of a left THA for periprosthetic fracture. Two months prior she had suffered a dislocation that had been left unreduced. Radiographs demonstrated that the implant was still dislocated at the time of presentation and that there was shell loosening (Figure 2D). Intraoperative findings included a grossly loose shell with a 15° elevated acetabular liner rim. The liner had embedded debris and impingement damage that was less than 1 mm deep (Grade 2, HSS scale).

*Head E*. A 43-year-old male presented five years post THA with a history of one early postoperative dislocation and four recent dislocations which had occurred within a four-day interval. The liner impingement damage was less than 1 mm deep (Grade 2, HSS scale). 

Light interferometry optical profilometry (OP) was conducted to capture the topography of the surface features [7]. Smaller-area (1200 × 960 *μ*m) images were obtained using a 5x lens. Larger-area images (~2 × 2 mm) were created by stitching together multiple smaller-area images captured by a 20x lens. Uniform hemispherical curvature was removed from the raw scans using a best-fit sphere calculated using a least squares fit to the appropriate quadratic fitting algorithm [7]. The OP data were used to calculate average roughness (*R*_*a*_), the root mean square roughness (*R*_rms_), and the average of the ten highest peaks and ten lowest valleys (*R*_*z*_). These OP data were calculated within eight 2 × 2 mm regions of interest each within both the clean and deposition areas of each of the five heads. The total percentage of the deposit regions scanned ranged from approximately 15% (head C) to 68% (head A).

Scanning electron microscopy (SEM) was performed at an accelerating voltage of 10 kV and a typical working distance of ~10 mm using FEI/Philips Sirion Field Emission SEM. Energy dispersive spectroscopy (EDS) was used to estimate elemental compositions of 5-6 locations within the deposition areas of each head. Selected distinct particles observed on the deposit areas of the femoral heads were also analyzed via EDS mapping, using a resolution of 1024 × 800 pixels (each pixel = 67.8 × 67.8 *μ*m) and a dwell time of 200 *μ*s. True elemental compositional measurements were performed on a 1 × 1 mm area (marked with an asterisk in Figure 1) of the deposition observed on head A, in an ultrahigh vacuum environment (1.2 × 10^−7 ^Pa) using X-ray photoelectron spectroscopy (XPS) with a monochromatic Al K-alpha source (*H*_*v*_ = 1486.6 eV). This scan was performed from 0 to 1400 eV with a pass energy of 117.4 eV and an energy step of 1.0 eV.

## 3. Results

Each femoral head featured a large, noticeably dark region (Figure 1). Visually, head A showed a nearly featureless dark swath located close to the apex (Figure 1A). In head B, the main dark region was composed of many overlapping scrapes centered slightly below the equator (Figure 1B). Head C displayed a large dark zone also consisting of many overlapping scrapes; this region appeared much more diffuse, with individual features being difficult to discern (Figure 1C). Head D displayed a very dark region concentrated in a single discrete zone in the lower portion of the head (Figure 1D). Head E also had a dark region of overlapping scrapes (Figure 1E).

**Figure 3:**
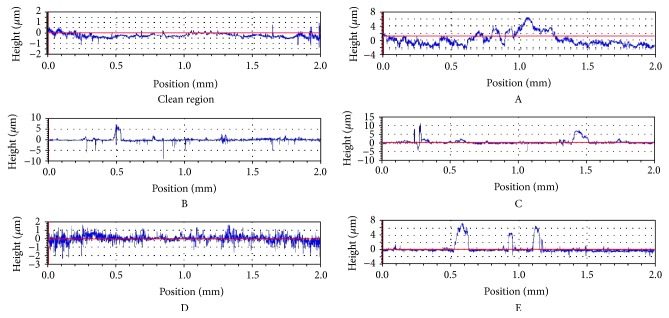
OP data from a “clean” region and from the deposit regions on retrieved femoral heads A–E.

Optical profilometry with roughness measurements illustrated the distinctions between the deposit and clean regions on the femoral heads (Figure 3). The deposit regions were associated with increased roughness (Table 1) and tended to involve many isolated peaks. Roughness increases between the clean and deposit regions on each head were 2.5–12.5, 4.0–11.7, and 5.0–10.8 for *R*_*a*_, *R*_rms_, and *R*_*z*_, respectively. Although individual *R*_*a*_ and *R*_rms_ values in the deposit regions were mostly at submicron levels, individual *R*_*z*_ values were generally more than 10 *μ*m for the deposit region.

**Figure 4:**
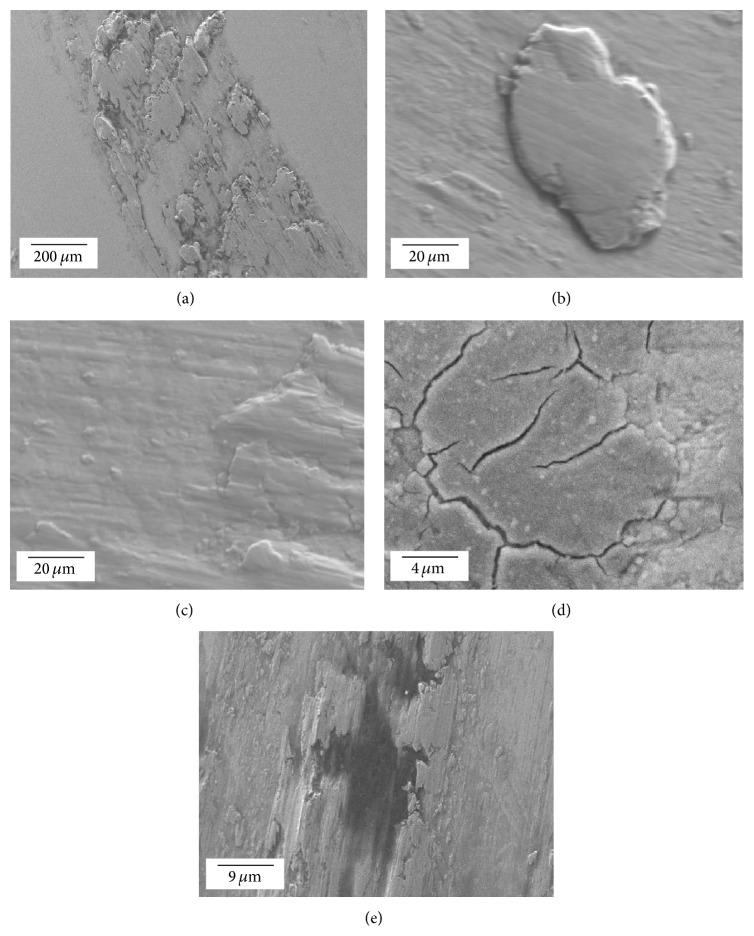
Representative microscopic morphological features observed by SEM on the deposit regions on the femoral head surfaces showing (a) pronounced scrape with deposits, (b) large, isolated deposits, (c) areas containing many small deposits <10 *μ*m in diameter, (d) otherwise relatively featureless film showing some evidence of fracture, and (e) evidence of more than one compound within the deposit region. Images (a)–(e) are, respectively, from femoral heads A–E in Figure 1.

Surface irregularities observed by SEM within the dark regions of the retrieved femoral heads ranged from submicron to multimicron sizes and exhibited a range of morphologies (Figure 4). These included large areas containing pronounced scrapes with deposits (Figure 4(a)), large deposits (>10 *μ*m in diameter) (Figure 4(b)), small particle deposits (<10 *μ*m in diameter) (Figure 4(c)), scaly films with fractures (Figure 4(d)), and contrast differences that suggested that more than one compound existed within a deposit region (Figure 4(e)). Sites with deposits standing proud to the surrounding topography were frequently observed in the dark regions. Fine-scale scratches were widely evident, but had no consistent direction.

**Figure 5:**
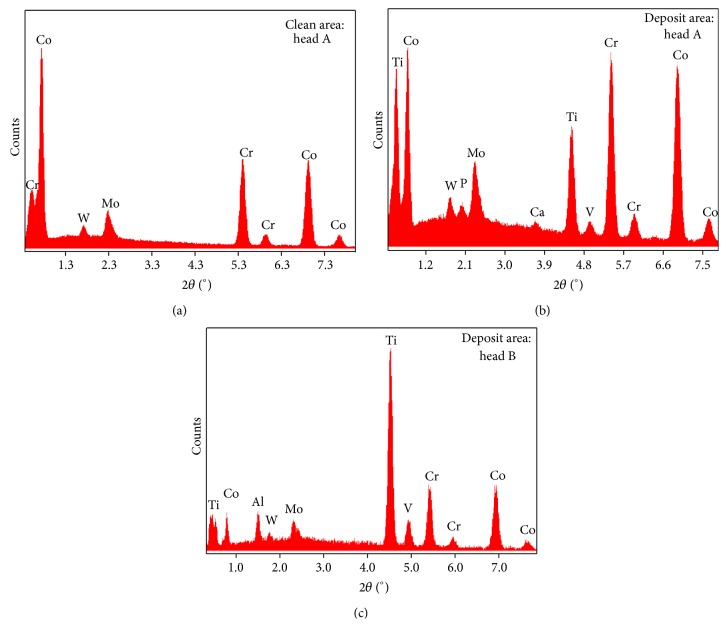
EDS data from a clean area on head A and from the deposit areas on heads A and B. The sites from which these data were collected are noted by asterisks (*∗*) in Figure 1. Similar data were collected from deposit areas of all heads (sites marked with *∗* in Figure 1), displaying peaks indicating the additional presence of bearing-foreign elements Ti, Al, V, Ca, and P.

**Figure 6:**
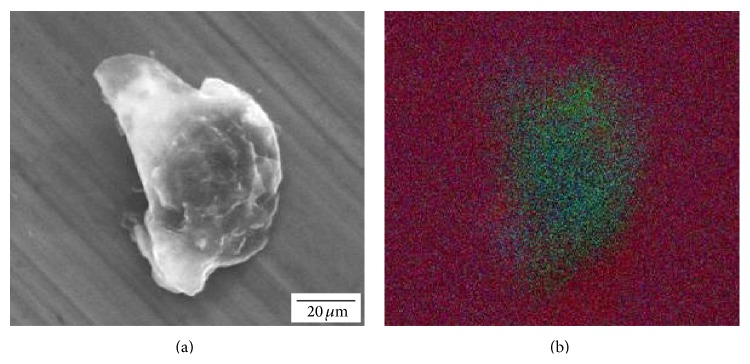
(a) SEM and (b) false-color EDS map of a tungsten-rich particle on the surface of head A. The EDS mapping demonstrates that these particles were compositionally distinct from the surrounding surface.

**Figure 7:**
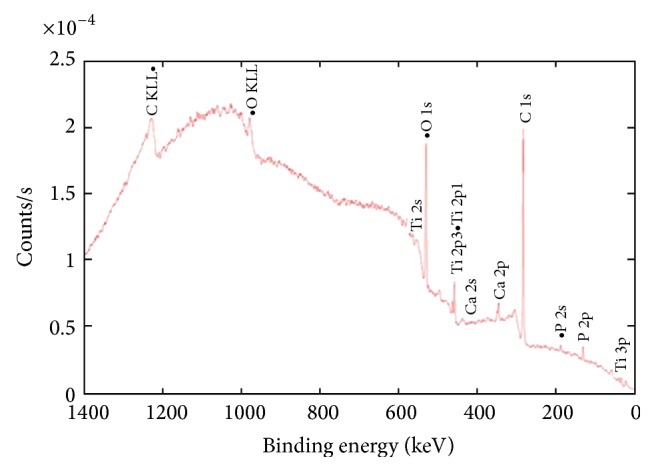
XPS data obtained from a 1 × 1 mm area in the deposit region on head A (site marked with an asterisk (*∗*) in Figure 1A), showing presence of impurities corroborating the data observed in EDS. Nominal surface compositions associated with this scan are O: 68.75%, Al: 3.07%, P: 9.39%, Ca: 8.13%, Ti: 7.01%, Co: 3.68%, Cr: 0.2%, and W: 0.03%.

The clean areas of the femoral heads had EDS peaks corresponding to Co, Cr, Mo, and W (Figure 5(a)). In the deposit areas, Ti, Al, V, Ca, and P also were found (Figures 5(b) and 5(c)). The intensity of the EDS peaks within the dark areas was highly variable. Compositionally distinct tungsten-rich particles (Figure 6) were observed on top of the deposits in all five heads. XPS performed on the deposit area on head A (Figure 7) confirmed the presence of Ca and P on the surface, with the deposit having a nominal metals basis composition of 22.2% Ti, 25.8% Ca, and 29.8% P.

## 4. Discussion

Retrieved cobalt-chrome femoral heads that had experienced dislocation or subluxation had deposit regions indicative of titanium transfer from the acetabular shell. The deposit areas were much rougher than the surrounding clean areas, and they displayed several different morphologies. Titanium alloy elements were the predominant constituents of the deposits. Calcium and phosphorous were also detected on top of the deposition surfaces, and tungsten-rich particles were detected within the deposit areas. The presence of calcium and phosphorus could indicate the presence of either stoichiometric or reduced hydroxyapatite. These deposition features could conceivably accelerate damage and wear of the opposing UHMWPE liner and thus accelerate the subsequent development of osteolysis [8–10].

Limitations of this study are the small number of heads analyzed and the modest numbers of areas analyzed on each head. These specific femoral heads were chosen on the basis of striking visual evidence of deposition and clinical histories indicating a likelihood of head-shell contact. Representative locations within the deposition areas were chosen for analysis since it was not feasible to analyze entire deposition areas.

Some of the maximum deposit peak heights found in the current study (5–60 *μ*m) were considerably larger than those observed previously on dislocated cobalt-chrome heads having suspected or confirmed material transfer: up to 10 *μ*m [11], 6.2 and 8.5 *μ*m [12], ~4.5 and ~7 *μ*m [13], and 1.6–4.3 *μ*m [14]. Generally similar average roughness for the deposit areas was noted in both the current study and in previous studies involving retrieved metallic femoral heads that had experienced head-shell contact. *R*_*a*_ of the deposit areas was 0.34 ± 0.11 *μ*m in the current study, in line with *R*_*a*_ values reported previously: 0.380 *μ*m [1], ~0.15–0.28 *μ*m (including scratches as well as transfer deposits) [14], 0.241 *μ*m (metal-on-metal THA) [15], and 0.338 *μ*m (metal-on-metal THA) [16]. Donaldson et al. reported a fivefold increase in *R*_*a*_ between clean and deposit areas, comparable to the *R*_*a*_ increases of 2.5–12.5-fold (an average of 4.6) found presently [17]. *R*_*z*_ of the deposition areas was 36.72 ± 22.46 *μ*m, considerably higher than the 1.13 *μ*m (maximum) reported in a previous metal-on-metal THA study [15].

EDS has frequently been used to detect the presence of titanium [1, 14–17] and aluminum [14, 15] in deposits on retrieved metallic femoral heads. Such deposits are obviously undesirable as they could accelerate bearing surface wear, a fact that has often been remarked upon qualitatively. However, recent developments in computational tribology [10] now make it possible to quantitatively link surface tribological aberrations with accelerated wear in individual cases. Hence, quantitative characterization of these surface aberrations and their linkage with clinical failure mechanisms has become of increased interest.

Calcium and phosphorus have been previously detected on retrieved heads from metal-on-metal bearings [18–21], although these elements were not associated with titanium deposition as was the case in the current study. Howie et al. suggested that the debris containing calcium, phosphorous, and oxygen that was found on the articulating surfaces and within wear tracks could be either from bone or from precipitated calcium phosphate [18]. McKellop et al. attributed surface deposits of calcium phosphate to precipitation from synovial fluid [19]. For the deposit analyzed with XPS in the current study, the apparent Ca/P ratio was close to unity, suggesting that these elements were in the form of brushite, which has been shown* in vitro* to act as a precursor to other calcium-phosphorous molecules [22, 23]. Spontaneous nucleation of calcium phosphates on titanium alloys* in vitro* is a well-established phenomenon [24–27], culminating in the formation of carbonated hydroxyapatite. Clinically, such a mechanism could eventually result in the formation of* de novo* abrasive third bodies. EDS has limited sensitivity to the presence of calcium and phosphorous as they are relatively lighter elements [28]. That these lighter elements were detected in this study with EDS at all suggests that the thickness of these secondary deposits was appreciable. Importantly, however, calcium and phosphorous were not detected on clean (nondeposit) areas in this group of specimens; thus, there was no evidence for generalized deposition of these elements. Build-up of “secondary” deposits of calcium and phosphorous could potentially increase head roughness (and hence, exacerbate polyethylene wear) beyond that occurring by “primary” titanium alloy deposits alone.

Tungsten-rich particles were observed within the deposition areas for all five femoral heads studied. Tungsten is not a constituent of orthopaedic titanium alloy, but it is a minor constituent (0.2% maximum) of ASTM F75 cobalt-chrome alloy [29], appearing in the microstructure as tungsten carbide (WC) particles that block the motion of lattice discontinuities [30]. To our knowledge, WC particles have not been previously reported as surface contaminants of retrieved total joint replacement implants. There have, however, been similar observations of other minor constituents of cobalt chrome alloy appearing in particulate deposits on retrieval surfaces. Raimondi et al. detected silicon and manganese in spherical particles (5–10 *μ*m diameter) found on the surface of retrieved cobalt-chrome femoral heads [31]. Silicon and manganese are also minor constituents of ASTM F75 cobalt-chrome alloy (both 1% maximum) [29]; Raimondi et al.'s disproportionately high detection of these elements in those particles was attributed to the particles having been microsegregated within the cast cobalt-chrome head and subsequently released [31]. Davidson suggested a mechanism by which particles such as carbides and nitrides could be released from the femoral head as the surface metal is worn [32]. The tungsten-rich particles detected in the present study were also likely derived from the microstructure of the femoral component. Such released particles obviously could contribute to the third-body burden.

## 5. Conclusions

Femoral heads from metal-on-polyethylene THA implants with a clinical history indicating contact with the acetabular shell displayed not only titanium alloy deposition, but also evidence of calcium, phosphorous, and tungsten. These deposition features could conceivably accelerate damage and wear of the opposing UHMWPE liner and thus accelerate the subsequent development of osteolysis. Quantifying this increased roughness provides useful information for the robust computational simulation and prediction of the correspondingly increased wear rate. Besides the inherent roughness of the deposition material* per se*, the presence of particles dominated by minor constituents of the underlying bearing surface material, and the presence of Ca and P atop the deposits, is suggestive of increased third body burden in both the present and the future. This analysis is a good starting point for further in-depth studies considering XPS observations reported in this study. Patients with suspected head-shell contact from any circumstance should therefore be closely monitored for wear or osteolysis.

## Figures and Tables

**Table 1:** Comparison of roughness parameters averaged over eight representative OP scans each in both clean and deposition regions of the femoral heads.

Head	“Clean” region			Deposition region		
	*R*_*a*_ (*μ*m)	*R*_rms_ (*μ*m)	*R*_*z*_ (*μ*m)	*R*_*a*_ (*μ*m)	*R*_rms_ (*μ*m)	*R*_*z*_ (*μ*m)
A	0.04 ± 0.00	0.06 ± 0.01	1.33 ± 0.57	0.50 ± 0.20	0.70 ± 0.30	14.40 ± 4.70
B	0.08 ± 0.00	0.10 ± 0.01	6.11 ± 3.00	0.20 ± 0.10	0.40 ± 0.10	53.90 ± 10.40
C	0.09 ± 0.00	0.12 ± 0.01	9.59 ± 5.14	0.30 ± 0.10	0.50 ± 0.10	48.20 ± 6.40
D	0.07 ± 0.00	0.10 ± 0.01	1.64 ± 0.68	0.40 ± 0.10	0.60 ± 0.10	10.40 ± 2.30
E	0.09 ± 0.02	0.13 ± 0.03	8.75 ± 5.45	0.30 ± 0.10	0.70 ± 0.20	56.70 ± 14.00
Average	**0.07 ± 0.01**	**0.10 ± 0.02**	**5.48 ± 4.89**	**0.34 ± 0.09**	**0.58 ± 0.19**	**36.72 ± 10.96**
